# American Medical Association

**Published:** 1860-09

**Authors:** 


### American Medical Association.

The following Editorial, from the "N. O. Medical News and Hospital Gazette," is so full of good sense and sound reasoning, and so nearly accords with our views, that we insert the article entire in lieu of what we might say upon the subject:

We occupy considerable space this time with the remainder of the report of the proceedings of the American Medical Association at the meeting lately held at New Haven, Conn., because we know all our readers will wish to know what was done there. Socially, no doubt, the doctors had a good time of it, but the abstract of proceedings does not enable us to form any estimate of the intellectual and scientific feast spread before them. Only when the volume of reports comes forth from the press can any one give an opinion. We are sorry to see that only three essays were handed in, and that not one of these was deemed worthy of the prize. If we are not mistaken, this is twice in succession that the prize has been withheld. This is rather a severe commentary. It shows conclusively, to our mind, a want of interest in the Association on the part of some of the best men in the profession. Nor do we wonder at this. As matters now stand, we fear the interest will diminish from year to year, and the Association is in danger of diminishing into a mere traveling ventilator of the few individuals amongst whom it happens to hold its meetings. The first honor of the Association has become a mere bauble with which to tickle the old men of the cities in which the meetings are held. A man is not made President of the Association because of his pre-eminence in a professional point of view; on the contrary, this strongest and only legitimate claim is ignored, and age or personal local popularity urge him into the position. This is all wrong, and we fear the Association will find it out only when it is too late.

From the report of proceedings, the reader will perceive that, as usual, any amount of talk has been indulged in on the subject of "Medical Education." The Committee of the Association, through the Chairman, Dr. Reese, of New York, made a report and presented sundry labored resolutions, and the Committee of the School Convention presented its report and resolutions.—Then the Association discussed and passed the last named resolutions, with amendments; and if the reader can tell us what has been really done in the premises, he can do more than we can. We have ever been the earnest advocates of reform in medical teaching, but ours is a practical, a tangible reform, for which we are laboring every day in the year. Reports and resolutions will never educate young men properly. The Association should point to a reasonable scale of progress—one to be extended as our country rises in civilization; then, with an unprejudiced eye, watch the schools and see where the greatest effort to effect the greatest amount of good is being made, and upon such heap the whole moral weight of which it is possessed. As it is, it does not require a Solomon to see that things are out of joint. That busy mole, Selfishness, is at work, and he must be caught and strangled.

Resolution No. 1 is neither more nor less than an attempt at immolation of the summer schools. There is no argument against the system of summer education; there is no proof of such schools being unable to impart proper instruction to pupils; there is nothing but a sweeping blow at their prosperity, and that blow comes really from those who teach in winter.—We are no champions of the summer schools, but we see plainly that this blow is unjustly dealt, and that the Association has allowed itself to be made a cat's paw of in the business. The object will not be attained, and the Association will have her escutcheon stained.

Resolution No. 2 proposes only what all well regulated medical schools do already. No. 3 requires not less than three or four months of "hospital clinical instruction." What constitutes such "instruction?" If it is only meant to have a building full of beds, with an amphitheatre for "clinical lectures," then the devil is easily whipped around the stump, and every little inland school can come up to the requirement. But if it is required that the beds shall be filled with sick men, women, and children, and that the student shall be carried from bed to bed, and be well drilled in all that pertains to the *practice* of medicine and surgery in all its various phases, then good bye to the vast majority of the schools in this country, for they must surely wither under the frown of the Association. Even the Philadelphia schools must give up the ghost, for we do know that she cannot furnish this species of instruction to her pupils. Indeed, we have had opportunities for personal observation, and we know that no city except New Orleans can come up to the proper standard—viz: *daily bedside observation of disease in all its forms*.

Resolutions Nos. 4 and 5 provide for the attendance of State delegates at the school examinations for the degree of M. D.— Yet there cannot be a sane man who does not know that outside some little State about the size of one of our Southern counties, and for any other school than one of eight or ten graduates, there could not be found the *proper* men to undertake such a task. *Proper* men would have to be well paid for the trouble and loss of time, and who is to pay them? Of course a herd of martyrs to the great cause could be found amongst those whose lives are devoted to picking the motes from the eyes of their fellow-men, while their own eyes require sweeping. Such men will never control the schools of this country. They may spout reports and resolutions, but there their occupation ends.

Resolution No. 6 says that no college shall be recognized which does not require evidence of suitable preliminary education from all applicants for instruction. What constitutes the "suitable instruction?" Far be it from us to apologise for, or to foster ignorance; but the plainest dictates of common sense point us to the fact that many men with very limited preliminary education, must, through a long series of years yet, be admitted into our medical schools.

A great portion of our country is yet in the condition which renders it impossible for all our young men to acquire that high degree of education which fits the physician for being most useful, and at the same time ornamental to society. In the plain nature of things, it is imposaible that the "suitable education," can be had by all. Shall we then refuse our less fortunate fellow-men of the less enlightened localities the knowledge they seek at our hands because they cannot make the *best* use of it?— Their people must have doctors; those who have had better opportunities will not go amongst them, and shall we refuse to give them all the information we can—all they are able to seek at our hands? Wise authors of wise resolutions! It is time, gentlemen, that you were giving the world some practical demonstration of the *great interest* you feel in the cause of medical education, and you cannot do it half so well by spouting resolutions, as by going amongst our frontier people and saving them from the men you would deny instruction.

Resolutions 7, 8 and 9 are about as forcible as the foregoing. Sum them up, reader, and tell us what it all amounts to.

Again, we say, the power of the American Medical Association is a great moral power, which, if judiciously exerted, can do infinite good. If the Association is properly composed, it is but the voice of the "people" of the profession; the members are but the mouth-pieces of the people of their respective districts. What then? Let this Association keep a watchful eye over the schools, and let each member, and each constituency of each membe, patronize only that school which is pressing onward. Then shall we see good results, and then only. Again, the Association *must* be like Cæsar's wife. She has a code of ethics, and she must see that no man violates it with impunity. As it is, it is ignored by too many whose voices are all the time ringing for improvement in their fellow-men. For ourselves, we frankly confess that we have within us an irrepressible repugnance to being either led or driven by men whose every act but shows them to be either selfish or self-sufficient. There is real pleasure in lending ourselves to the accomplishment of a great and general good; but it is degrading to use so good a cause as a cloak under which to conceal a poisoned dagger for even the humblest of our fellow-men.

Let us have a steady and reasonable reform in the system of medical education, but let that reform be the fruit of co-operation between the schools and the members of the American Medical Association, who not only faithfully adhere to her code of ethics, but who stand ready to defend it against all who dare to use it for unholy purposes. This is what every good and true man in the profession wishes, what every good man stands ready to enforce. We have ever defended the American Medical Association from its enemies, and we are more than willing to acknowledge her as the great head of the profession of this country, but has no arbitrary powers, and can never have any; she has the right to say that a medical man *ought* to know how to read Latin, but she has no right to say that he *shall* know it. She has a right to bestow her highest honors on the most highly educated and ablest men in the profession, but she has no right to say that an individual inferior in each point of view shall have no seat in her hall. The people who live under a republi can form of government, and know no similar power in any of its branches, will surely revolt if it is attempted to be enforced on them.

